# Comparative Evaluation of Bioactive Compounds, Antioxidant Capacity, and Quality Traits of Raspberry Cultivars

**DOI:** 10.3390/molecules31203703

**Published:** 2026-10-15

**Authors:** Kamil Buczyński, Magdalena Kapłan, Tomasz Czernecki, Anna Stój

**Affiliations:** 1 Institute of Horticulture Production, University of Life Sciences in Lublin, Głęboka 28, 20-612 Lublin, Poland; magdalena.kaplan@up.edu.pl; 2 Department of Biotechnology, Microbiology and Human Nutrition, University of Life Sciences in Lublin, Skromna 8, 20-704 Lublin, Poland; tomasz.czernecki@up.edu.pl (T.C.); anna.stoj@up.edu.pl (A.S.)

**Keywords:** *Rubus*, fruit quality, phenolic, anthocyanins, flavonoids

## Abstract

Although raspberries are widely valued for their health-promoting properties, comprehensive comparative studies integrating pomological quality traits, phenolic compounds, and antioxidant capacities across cultivars with distinct fruit colors remain sparse. Sixteen raspberry cultivars, including 12 red-fruited, three yellow-fruited, and one cultivar with black-purple fruits, were evaluated at a single location during one growing season. This study evaluated the physicochemical parameters, bioactive compounds, antioxidant activity, and selected phenolic acids of red-, yellow-, and black-purple-fruited raspberry cultivars grown under the same environmental conditions. Antioxidant capacity was determined using FRAP and DPPH assays. The results revealed highly significant cultivar-dependent variations in dry matter, total soluble solids, titratable acidity, TSS/TA ratio, vitamin C, tannins, total phenolics, flavonoids, anthocyanins, and overall antioxidant performance. The black purplefruited cultivar stood out with exceptionally high levels of total phenolics, flavonoids, anthocyanins, and FRAP activity. Red cultivars exhibited wide phenotypic and biochemical variability, while yellow cultivars displayed minimal anthocyanin accumulation. Correlation analysis highlighted a strong relationship between antioxidant potential (FRAP) and flavonoid content, alongside moderate correlations with anthocyanins and vitamin C. Most targeted phenolic acids were either not detected or occurred at concentrations below the limit of quantification, with only a few quantifiable detections. These findings provide insight into cultivar-dependent variation in raspberry fruit quality and bioactive composition and may support the selection of cultivars for further evaluation according to specific quality traits.

## 1. Introduction

The genus *Rubus*, recognized for its considerable taxonomic complexity [1], represents the most species-rich genus within the family Rosaceae, comprising, depending on the source, from approximately 500 to over 1400 species [2,3,4,5,6,7]. Species of the genus Rubus are predominantly distributed in temperate regions, but they also occur in subtropical and Arctic zones [8]. These plants are utilized worldwide both as food sources and for medicinal purposes [9]. Berry species are characterized by unique nutritional profiles, encompassing a wide range of bioactive compounds and phytochemicals [10,11]. This has been reflected in numerous studies conducted in recent years, which have confirmed multiple health-promoting effects associated with berry consumption, thereby contributing to the growing trend in their intake [12]. The extensive bioactivity exhibited by *Rubus* species underscores the need for continued exploration and utilization of these plants in both dietary and medicinal contexts. The genus *Rubus* represents a valuable resource for improving human health, and its expanded cultivation and utilization could significantly contribute to global health and nutrition [13]. The commercial utilization of the genus *Rubus* is presently confined to a limited number of cultivated species, whereas many non-traditional and wild edible species remain largely underexploited [14].

For local market production, three main raspberry species are cultivated: red raspberry (*Rubus idaeus* L.), including yellow-fruited variants, eastern black raspberry (*Rubus occidentalis* L.), and the interspecific hybrid (*Rubus × neglectus* Peck). Increasing the diversity of raspberry fruit colors available on the market may offer benefits for both consumers and producers [15]. The production of black raspberry (*R. occidentalis*) has been primarily concentrated in western Oregon, with recent expansion observed in South Korea. Purple raspberries, resulting from hybridization between red and black raspberries, are cultivated on a limited scale across North America and in northeastern China. In contrast, Arctic raspberry (*Rubus arcticus* L.) and cloudberry (*Rubus chamaemorus* L.) are predominantly harvested from wild populations in Scandinavian regions. Red raspberry (*R. idaeus*) is grown mainly in Europe and North America [16], and in the future, China may become one of the main growing regions for this species [17,18].

The identification of new cultivars exhibiting traits desirable to growers, processors, and consumers remains an ongoing effort. As scientists continue to develop novel varieties using both conventional and molecular approaches, the evaluation of these cultivars in terms of their nutritional value remains a significant challenge [10]. Raspberry cultivars can be classified into floricane and primocane types. Floricane cultivars do not bear fruit during the first season of cane development; in the second year, they produce lateral fruiting branches from dormant buds, after which the canes senesce following harvest. In contrast, primocane cultivars develop fruiting laterals on the upper portions of current-season canes. The lower portions of these canes also form dormant buds, which may give rise to fruiting branches in the following year, similarly to floricane types. This characteristic enables primocane cultivars to be managed for a double-cropping system, where fruit is first produced on canes retained from the previous season, followed by fruiting on canes developed during the current growing season.

Raspberries are gaining increasing recognition for their culinary versatility as well as their broad range of applications. This trend is likely driven by growing consumer awareness of health and wellness, alongside the expanding body of scientific research and media coverage highlighting their unique nutrient and phytochemical composition and their potential role in reducing disease risk [19]. From an economic perspective, fruits of the genus Rubus may represent promising raw materials for the development of value-added food products with enhanced nutritional and functional characteristics [20].

Phytochemical analyses have demonstrated that raspberries are a rich source of bioactive compounds, including phenolics, terpenoids, alkaloids, steroids, and fatty acids, with approximately 194 compounds identified to date [21]. Furthermore, numerous pharmacological activities have been reported, including hypoglycemic and hypolipidemic effects, as well as anti-Alzheimer’s, anti-osteoporotic, hepatoprotective, anticancer, neuroprotective, antibacterial, and skin-protective properties [13,22,23,24,25].

Raspberries exhibit high antioxidant and anti-inflammatory activity, which may be beneficial for patients with chronic diseases. The family of phenolic compounds present in raspberries may serve as a potential adjuvant to reduce oxidative stress and inflammation in individuals with chronic conditions, as well as a prophylactic therapeutic approach [26]. In the future, there is a clear need to strengthen research on *Rubus* berries in the fields of phytochemistry, pharmacology, and functional foods in order to establish comprehensive quality control methodologies, dynamically monitor changes in active constituents during growth and processing stages, conduct in-depth pharmacological and pharmacodynamic studies, develop spectrum–effect relationship networks, and expand the range of applications of *Rubus* berries [27].

Studies also highlight the nutritional value of raspberries, attributed to their richness in minerals, polyphenols, and flavonoids. These findings emphasize the importance of incorporating these fruits into the daily diet to fully benefit from their nutritional properties. In particular, black raspberries stand out due to their higher nutrient content compared to red raspberries. From a practical perspective, regular consumption of these fruits should be promoted, with particular emphasis on the benefits of black raspberries. Furthermore, the food industry could explore innovative approaches to incorporating raspberries into a variety of products, thereby providing consumers with healthier options. Such initiatives may support dietary choices tailored to individual taste preferences and nutritional needs, ultimately promoting a healthier lifestyle [28]. The development of new raspberry cultivars is driven by the need to improve fruit quality, diversify product assortments, and increase yield. However, these advantages can be fully realized only under optimal interactions between genotype and agroecological conditions, reflecting the adaptability of a given cultivar [29].

Although raspberries are widely recognized as valuable sources of bioactive compounds, comprehensive comparative studies combining fruit quality parameters, phenolic composition, antioxidant activity, and selected phenolic acids in cultivars differing in fruit colour and fruiting type are still scarce. Therefore, the aim of this study was to assess the variability in physicochemical traits, bioactive compounds, antioxidant potential, and selected phenolic acids in raspberry cultivars grown under the same conditions. This approach was intended to provide a broader understanding of cultivar-dependent differences in fruit quality and to support the selection of genotypes with desirable sensory and health-promoting properties.

## 2. Results and Discussion

Fruit quality parameters, including dry matter, total soluble solids (°Brix), titratable acidity, and the TSS/TA ratio, are presented in Table 1. Significant differences among cultivars were observed for all evaluated parameters.

Dry matter content varied significantly among cultivars, ranging from 8.36 to 17.29%. The highest value was recorded for Delniwa, whereas Poranna rosa exhibited markedly lower dry matter compared to the other cultivars. Lower values were also observed for Heban and Glen Ample, while most of the remaining cultivars showed intermediate levels.

It should be noted that dry matter content may vary depending on the harvest period [30]. Sweetness and acidity are primarily determined by sugar and organic acid content, respectively. Their contribution to overall flavor depends not only on their concentrations but also on the specific types and relative proportions of sugars and acids present [31].

Total soluble solids differed significantly between cultivars, with values ranging from 8.50 to 13.30 °Brix. The highest total soluble solids values were found in Promyk, Delniwa and Husaria, whereas the lowest were recorded for Glen Carron and Glen Mor.

Titratable acidity ranged from 1.41 to 2.74 g/100 g FW. The highest acidity was observed in Poranek and Polana, while the lowest value was found in the black-purple cultivar Heban. Lower acidity values were also recorded for Polonez and Enrosadira.

The TSS/TA ratio showed considerable variation, ranging from 3.40 to 8.37. The highest ratio was observed in Promyk, followed by Polonez and Husaria. The lowest values were recorded for Polana, Poranek, and Glen Mor. Total soluble solids and titratable acidity are commonly used as key selection criteria for fruit flavour in breeding programs, as the perception of a well-balanced taste depends on the balance between sweetness and acidity [32]. Additionally, the TSS/TA ratio has been proposed as a quick measure of consumer acceptance of fruit flavor [33].

In studies on primocane- and floricane-fruiting raspberry cultivars with red, black, and yellow fruits (Bristol, Litasz, Benefis, Laszka, Radziejowa, Sokolica, Willamette, Polka, Polana, Polesie, and Poranna Rosa), total soluble solids ranged from 7.1 to 13.2 °Brix, while titratable acidity varied between 1.2 and 2.7%. For the cultivars Polana, Polesie, and Poranna Rosa, depending on the study year, total soluble solids respectively ranged from 11.6 to 12.5, 12.2 to 12.5, and 10.8 to 12.7 °Brix, while titratable acidity ranged from 2.3 to 2.4, 2.0 to 2.1, and 2.2 to 2.7% [34].

In other studies, lower dry matter content for Glen Ample (10.53%) was reported, whereas total soluble solids (8.90 °Brix) and titratable acidity (1.94%) were comparable [35]. Comparable dry matter content for the cultivar Polesie has been reported in previous studies, approximately 14.3%, whereas lower values were observed for Polana (13.2%) [36].

Studies on 24 red raspberry cultivars reported titratable acidity ranging from 4.90 to 17.51 g/100 g DW and total soluble solids from 6.33 to 14.33 °Brix [37]. Studies on red raspberry cultivars (Heritage, Summit, Harvest, red Autumn Bliss) and the cultivar yellow Autumn Bliss reported titratable acidity ranging from 1.82 to 2.00% and total soluble solids from 7.00 to 11.00 °Brix [38]. The variability in sugar content and acidity among fruits of the genus *Rubus* enables their utilization across different sectors of the agri-food industry [39].

The observed variability in fruit quality parameters among raspberry cultivars may reflect differences in metabolic allocation between primary and secondary metabolites, which may contribute to differences in fruit quality and composition. Higher total soluble solids and lower acidity may contribute to a sweeter or more balanced flavour profile, although consumer acceptance was not directly evaluated in the present study. However, increased dry matter content may indicate a higher concentration of structural and bioactive compounds. These patterns suggest that fruit quality is shaped by complex interactions between sugar accumulation, organic acid metabolism, and the synthesis of secondary metabolites, which may differ substantially among cultivars. Such variability may also reflect breeding strategies and genetic backgrounds, as well as adaptation to specific environmental conditions, highlighting the importance of cultivar selection depending on the intended use, whether for fresh consumption or processing.

The content of vitamin C and phenolic compounds, including tannins, and total phenolic compounds, flavonoids, and anthocyanins, in raspberry cultivars is presented in Table 2. Significant differences among cultivars were observed for all analyzed parameters. Vitamin C content varied significantly among cultivars, ranging from 6.53 to 32.01 mg/100 g FW. The highest value was recorded for Przehyba, while Heban showed the lowest level.

Tannin content differed significantly, with values ranging from 0.017 to 0.091% FW. The highest level was observed in Glen Carron, whereas Enrosadira showed the lowest content. Previous studies confirm the possibility of using preparations isolated from raspberry fruits with high tannin content for antimicrobial purposes [40], which further expands the directions of use of this raw material.

The content of total phenolic compounds ranged from 46.18 to 128.10 mg CAE/100 g FW. The highest value was found in Heban, while the lowest was observed in Husaria.

Flavonoid content varied considerably among cultivars, ranging from 0.66 to 8.89 mg RE/100 g FW. The highest value was recorded for Heban, while Glen Ample exhibited the lowest level.

Anthocyanin content showed substantial variation, ranging from 1.27 to 71.22 mg/100 g FW. The highest level was observed in Heban, whereas Promyk exhibited the lowest content. The observed differences in anthocyanin content may be associated with fruit colour.

Previous studies have reported higher levels of polyphenols and anthocyanins in black raspberry cultivars compared to red and yellow ones. In contrast, vitamin C content has been reported to be higher in red and yellow raspberries and lower in black-fruited cultivars [41]. Higher phenolic content has been reported in yellow raspberries compared to red ones, whereas anthocyanins show the opposite pattern, being more abundant in red fruits. In contrast, flavonoid content does not appear to be associated with fruit colour [38].

Other results were shown in the case of studies covering red raspberry cultivars Polana, Polka, Polesie, Benefis, Laszka, Radziejowa, Sokolica and Willamette, yellow raspberries (Poranna Rosa) and black raspberries (Bristol and Litacz), where yellow raspberry was characterized by values for phenolics depending on the season in the range of 182.58–406.02 mg/100 g FW, then red raspberry cultivars covered the range of values from 175.90 to 549.02 mg/100 g FW, with the highest values for black fruit cultivars 541.87–1351.59 mg/100 g FW. For anthocyanins, the highest values were also obtained for black raspberries (285.00–608.24 mg/100 g FW), followed by red raspberries (29.69–81.13 mg/100 g FW) and the lowest for yellow raspberries (5.87–20.39 mg/100 g FW). The authors also indicated that primocane-fruiting cultivars do not differ significantly from floricane-fruiting ones in terms of total phenolics, anthocyanins, and antioxidant capacity, suggesting that the fruiting type does not compromise fruit quality [42]. In studies involving 19 raspberry cultivars with red, yellow, and black fruits, the highest levels of phenolics and anthocyanins were also observed in black raspberries. For the cultivars Polana and Polesie, anthocyanin content reached 83.5 and 113.6 mg/100 g FW, respectively, while for the black raspberry cultivar Bristol it was 325 mg/100 g FW, and for the yellow cultivar Beglianka it was 2.1 mg/100 g FW [36].

Previous studies also indicate that the lack of anthocyanins in yellow-fruited cultivars is not accompanied by marked changes in other phenolic compounds [43]. Anthocyanin content is strongly associated with raspberry fruit colour, with low levels in yellow cultivars and high levels in black raspberries. Black raspberries have been reported to exhibit the highest anthocyanin content and antioxidant capacity among the studied genotypes. In red raspberries, higher anthocyanin content tends to be accompanied by higher total phenolic content and antioxidant capacity, whereas this relationship is not observed in yellow cultivars [44]. It has been suggested that breeding programs for fresh market red raspberries have focused on reducing anthocyanin content and fruit acidity in response to consumer preferences [45].

For seven wild raspberry (*R. idaeus*) genotypes, the following ranges were reported: phenolics (24.29–38.71 mg GA/g extract), flavonoids (4.39–4.97 mg Ru/g extract), tannins (0.20–0.45 mg/mL extract), and anthocyanins (0.31–4.73 µg/mL extract) [46]. For flavonoids, values were shown for the Tulameen (1.98 mg RUE/g) and Polka (2.16 mg RUE/g) varieties [47].

The observed variability in vitamin C and phenolic compounds among raspberry cultivars may indicate differences in the regulation of primary and secondary metabolic pathways. In particular, the marked variation in anthocyanin content, closely associated with fruit colour, suggests differential activity of the phenylpropanoid and flavonoid biosynthesis pathways. Since anthocyanins represent only a specific branch of the broader phenolic metabolism, their absence or low concentration in yellow cultivars does not necessarily indicate a reduced overall phenolic content, but rather a shift in the metabolic flux towards other phenolic compounds. This may explain why substantial levels of total phenolics can still be observed in cultivars with low anthocyanin content.

Antioxidant activity of raspberry cultivars, determined using FRAP and DPPH assays, is presented in Table 3.

FRAP values varied significantly among cultivars, ranging from 9.42 to 30.58 µmol Fe^2+^ eq./g FW. The highest value was recorded for Heban, while Delniwa showed the lowest level. Most of the remaining cultivars exhibited intermediate FRAP values. DPPH radical scavenging activity also differed significantly, ranging from 3.13 to 30.04 µmol TE/g FW. The highest activity was observed in Husaria, whereas Delniwa exhibited the lowest value. The majority of cultivars were characterized by moderate antioxidant activity.

Previous studies have indicated higher FRAP values in black raspberry cultivars compared to red and yellow ones. In contrast, DPPH activity was highest in red and yellow raspberries and lowest in black cultivars [41]. In studies involving cultivars of *R. occidentalis* and *R. idaeus*, as well as their hybrids, DPPH values for the cultivars Jewel, MacBlack, Niwot, Heban, Delniwa, and Husaria were 1.82, 1.63, 1.59, 1.53, 0.97, and 1.10 mmol TE/g DW, respectively, while FRAP values were 3.21, 3.56, 2.73, 2.47, 1.05, and 1.49 mmol TE/g DW. In terms of anthocyanins and polyphenols, red raspberry cultivars showed lower values than black raspberries and their hybrids [48].

The discrepancies observed between FRAP and DPPH assays may further highlight the complexity of antioxidant evaluation in raspberry matrices. Although both methods are commonly used to assess antioxidant activity, they rely on different reaction environments and may preferentially respond to distinct groups of compounds. This suggests that the antioxidant potential of raspberry fruits cannot be fully described by a single analytical approach, but rather reflects a combination of responses depending on the chemical composition of the sample. Therefore, the use of multiple assays provides a more comprehensive characterization of antioxidant properties and may better capture the functional diversity of bioactive compounds present in raspberry fruits.

This is consistent with previous reports indicating that no single assay can fully reflect the antioxidant capacity of complex systems and that multiple methods are required to obtain a more comprehensive evaluation [49,50,51,52].

Furthermore, differences in antioxidant activity among the raspberry cultivars may be due to the occurrence of carotenoid complexes that remain insufficiently identified and known [48].

Table 4 presents the mean fruit quality parameters, bioactive compound contents, and antioxidant activity of red-fruited raspberry cultivars grouped according to fruiting type. Significant differences between the groups of floricane- and primocane-fruiting cultivars were observed for most of the analyzed parameters. Floricane cultivars exhibited significantly higher contents of vitamin C, tannins, and total phenolic compounds, as well as higher FRAP values. In contrast, primocane cultivars were characterized by significantly higher dry matter content, total soluble solids, TSS/TA ratio, and flavonoid content.

These results indicate distinct compositional patterns between the evaluated groups of cultivars; however, because the floricane- and primocane-fruiting groups consisted of different genotypes, the observed differences cannot be attributed to fruiting type alone. Primocane cultivars appeared to be characterized by traits potentially related to higher sweetness and sensory acceptance, as indicated by their higher total soluble solids and TSS/TA ratio. Conversely, the floricane cultivar group showed higher levels of several bioactive constituents and greater ferric-reducing antioxidant activity, which may indicate a stronger contribution of vitamin C, tannins, and total phenolic compounds to their antioxidant profile. No significant differences between the two cultivar groups were observed for anthocyanin content or DPPH activity. Therefore, the observed differences should be interpreted as patterns among the specific cultivars included in each group, because genotype and fruiting type effects cannot be separated in the present experimental design, and should not be generalized as inherent characteristics of floricane- and primocane-fruiting raspberry cultivars. 

**Figure 1:**
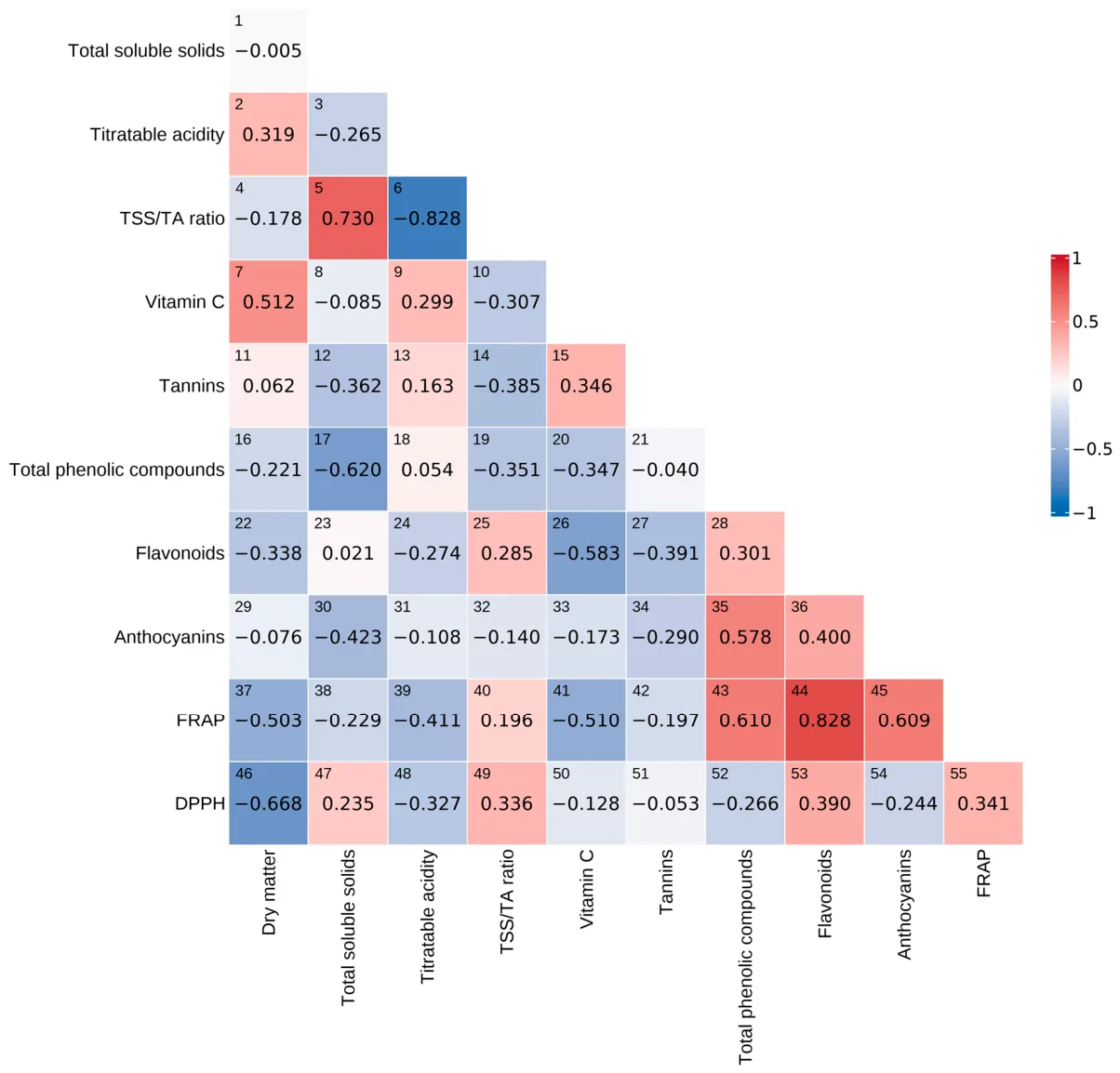
Parameter correlations.

The correlation heatmap indicated several associations among the studied parameters (Figure 1). Higher vitamin C content was associated with higher dry matter content, whereas higher flavonoid content was associated with lower vitamin C content. Higher anthocyanin content was associated with lower total soluble solids and with higher total phenolic content and flavonoid content. Higher FRAP values were associated with lower dry matter content, titratable acidity, and vitamin C content, but with higher total phenolic content, flavonoid content, and anthocyanin content. Higher DPPH activity was associated with lower dry matter content. Higher TSS/TA ratios were strongly associated with higher total soluble solids and lower titratable acidity. Because the TSS/TA ratio is mathematically derived from TSS and TA, correlations between these variables should be regarded as expected mathematical relationships rather than independent biological associations. In addition, higher FRAP values were strongly associated with higher flavonoid content. The observed associations suggest that antioxidant capacity is not governed by a single compound group but rather reflects the combined contribution of multiple bioactive constituents. In particular, the strong correlation between FRAP values and flavonoids may indicate that flavonoids play a major role in determining the reducing antioxidant capacity of raspberry fruits. This is consistent with the well-documented ability of flavonoids to donate electrons and hydrogen atoms, thereby scavenging free radicals in redox-based systems [53].

At the same time, moderate correlations of FRAP with anthocyanins and vitamin C may further support the notion that different classes of compounds collectively contribute to antioxidant activity. The involvement of vitamin C, although less pronounced than that of flavonoids, may reflect its role as a low-molecular-weight antioxidant that can interact with phenolic compounds and participate in redox cycling. These findings may therefore suggest a synergistic effect of various antioxidants present in raspberry fruits. Previous studies suggest that bioactive compounds in berries, including phenolics, contribute to antioxidant activity both individually and in combination, and may act additively or synergistically [11].

In contrast, the weaker and less consistent correlations observed for DPPH activity may reflect differences in the mechanisms underlying the applied assays. While FRAP is based on electron transfer, DPPH involves radical scavenging, which may depend on the structural characteristics and reactivity of individual compounds. This may indicate that not all phenolic compounds contribute equally to different types of antioxidant activity, and that the interpretation of antioxidant potential should consider the methodological approach used. Such differences between antioxidant assays have been widely reported and are attributed to variations in reaction mechanisms and the nature of radical or oxidant sources [54].

The observed relationships among anthocyanins, flavonoids, and total phenolic compounds may reflect their shared biochemical origin within phenylpropanoid and flavonoid metabolism, although the present analysis does not allow specific regulatory mechanisms to be established. This is consistent with previous reports indicating that flavonoids and their derivatives represent a major group of plant secondary metabolites synthesized through defined biosynthetic pathways [55,56].

Additionally, the moderate association between vitamin C and dry matter content, as well as between FRAP and dry matter, may suggest that fruit compositional traits are linked to the accumulation of bioactive compounds. Higher dry matter content may be associated with a greater concentration of soluble and structural components, including antioxidants, although this relationship may also depend on cultivar-specific factors and growing conditions.

The strong correlations observed between the TSS/TA ratio and its components confirm that this parameter primarily reflects the balance between soluble solids and titratable acidity rather than independent biochemical processes. This supports its common use as an integrative indicator of fruit taste quality rather than a direct marker of bioactive compound composition.

The present study has several limitations that should be considered when interpreting the results. The assessment of antioxidant activity was based on in vitro assays, which may not fully reflect biological activity in vivo. In addition, the applied extraction procedures primarily target free forms of compounds, potentially underestimating the contribution of bound or conjugated phenolics. Because the experiment was conducted at a single site during a single growing season, genotype × environment interactions could not be assessed. Consequently, the observed differences among cultivars should be interpreted as specific to the environmental conditions of the present study and should not be assumed to remain stable across different locations or seasons. Furthermore, the study focused exclusively on fruit samples and did not include other plant organs, such as roots, shoots, or leaves, which may also represent important sources of bioactive compounds. The analysis was limited to selected raspberry cultivars and did not include black raspberry genotypes. Moreover, the study did not address the content of macro- and microelements, which may also contribute to the nutritional value of raspberry fruits.

To complement the broader assessment of phenolic compounds, six selected phenolic acids were included as a targeted screening to determine whether they occurred at quantifiable levels in the studied raspberry cultivars under the applied extraction conditions. Their occurrence is presented in Table 5. 4-Hydroxybenzoic, caffeic, vanillic, and syringic acids were not detected in most cultivars. When detected, 4-hydroxybenzoic, caffeic, and vanillic acids occurred at concentrations below the limit of quantification (LOQ), whereas syringic acid was quantified only in Jantar (0.50 µg/g FW). Ferulic acid was detected in 15 of the 16 cultivars but was quantifiable only in Enrosadira (0.70 µg/g FW); it was not detected in Poemat. Sinapic acid was detected in all cultivars, with quantifiable concentrations in Polana, Poranna Rosa, and Heban (0.50 µg/g FW in each), whereas its concentrations in the remaining cultivars were below the LOQ. The low levels or lack of detection of phenolic acids in the analyzed raspberry cultivars may partly reflect their occurrence in bound or conjugated forms; however, this possibility was not directly assessed in the present study, as no hydrolysis or fractionation procedure was applied. Previous studies using separate analysis of free and bound phenolic fractions have shown that alkaline hydrolysis can release phenolic acids from plant matrices, including p-coumaric, gallic, and caffeic acids [57]. In raspberry fruits, phenolic acids are generally considered minor constituents compared to other phenolic groups, such as anthocyanins and flavonoids, which typically dominate the phenolic profile. Therefore, their limited detectability may reflect both their relatively low abundance and methodological constraints related to extraction and quantification. Accordingly, this analysis should be regarded as a targeted assessment of selected phenolic acids rather than as a comprehensive characterization of the phenolic acid profile of raspberry fruits.

In previous studies on red raspberry cultivars, citric acid was identified as the predominant organic acid in all varieties, followed by malic acid. Oxalic and α-ketoglutaric acids were detected in all varieties at low levels, while tartaric, lactic, fumaric, and succinic acids were present only in some varieties. Fumaric acid occurred in trace amounts [37]. In other studies, ellagic acid was identified as the predominant phenolic compound, followed by gallic, chlorogenic, caffeic, and p-coumaric acids. Lower concentrations were observed for catechin, rutin, quercetin-3-β-D-glucoside, protocatechuic acid, quercetin-3-glucuronide, and hydroxybenzoic acid [38]. Studies focusing on *R. idaeus* fruits have shown the following values for individual hydroxobenzoic acids, gallic acid (0.194), vanillic acid (0.025), syringic acid (0.012), 3-hydroxybenzoic acid (0.019), ellagic acid (0.007), protocatechoic acid (0.008), in the case of hydroxycinnamic acids chlorogenic acid (0.391), caffeic acid (0.011), methoxycinnamic acid (0.023), ferulic acid (0.033), coumaric acid (0.007) and cinnamic acid (0.027). Moreover, the authors demonstrated that hydroxybenzoic acids exhibited the highest bioaccessibility in the intestinal phase, highlighting the importance of bioavailability for the potential health effects of raspberry fruit compounds [58]. Studies on the content of salicylic acid in raspberry fruits have not revealed its presence [31].

Other studies on *R. idaeus* fruits did not detect any content of p-Hydroxybenzoic acid, gallic acid, chlorogenic acid, and o-Coumaric acid, but confirmed the presence of caffeic acid (139.95), p-Coumaric acid (66.11), ferulic acid (467.26), m-Coumaric acid (339.71), and vanillic acid (18.66). The authors suggested that insoluble-bound fractions of berries represent a significant source of natural antioxidants and may contribute to the prevention of oxidative stress–related diseases [59].

The quality of raspberry fruit is strongly influenced by both genotype and harvest season, with genotypes responding differently to interannual variability. Consequently, the importance of multi-year evaluation of new genotypes under experimental conditions is emphasized in order to identify those combining high nutritional value with stable fruit quality across varying seasonal conditions [60]. Previous studies highlight considerable variability in antioxidant composition among raspberry cultivars, which may influence their antioxidant properties. This variability suggests considerable potential for the selection and development of cultivars with desirable phytochemical composition and antioxidant properties. Further studies focusing on the biosynthesis and regulation of these compounds, as well as on the factors influencing their accumulation, may support breeding strategies aimed at producing raspberries with improved nutraceutical quality [61]. Significant advances have been achieved in raspberry breeding; however, these developments likely represent only an initial stage of future progress, particularly with the expanding use of germplasm resources conserved in repositories worldwide. At present, approximately 50 active raspberry breeding programs operate across 26 countries, mainly in Europe and North America. Contemporary breeders benefit from extensive germplasm collections, modern analytical tools, improved training, advanced breeding strategies, and the cumulative knowledge generated through more than a century of breeding efforts conducted by numerous researchers globally [16]. The importance of genetic diversity among raspberry cultivars extends beyond breeding purposes and may also contribute to dietary diversity and variation in the nutritional composition of raspberry fruits [62]. It should also be emphasized that environmental factors play an important role in shaping the chemical composition of berry fruits. In addition, the broader exploitation and cultivation of entire raspberry plants and their components under diverse growing conditions may provide new prospects for improving phytochemical composition and antioxidant potential [63]. Moreover, research indicates a high content of macro- and microelements in red, yellow, purple and black raspberries, which further increases their value [64]. But also in the case of nutritional values, their dependence on the color of the raspberry fruit is emphasized [65]. Species of the genus *Rubus* also provide valuable raw material in the form of leaves, which represent a rich source of bioactive compounds with potential health-promoting properties [66,67,68,69,70].

Overall, the present results indicate substantial variation in raspberry fruit quality and antioxidant properties among the evaluated cultivars under the conditions of the present study. The observed differences likely reflect a combination of cultivar characteristics, fruit composition, and environmental influences, which cannot be fully separated within the current experimental design. The relationships identified between phenolic composition and antioxidant capacity indicate that several classes of bioactive compounds are associated with the chemical antioxidant properties of raspberry fruits, rather than these properties being related to a single constituent group. This highlights the importance of considering the overall compositional profile when evaluating the bioactive composition and antioxidant properties of raspberry fruits.

From an applied perspective, these findings underscore the importance of cultivar selection as a key determinant of both sensory and bioactive compound composition. The observed diversity in biochemical composition suggests significant opportunities for the development of raspberry cultivars tailored to specific uses, including fresh consumption, processing, and functional food applications.

More broadly, the results emphasize the relevance of maintaining and exploiting genetic diversity in raspberry breeding programs, not only to enhance fruit quality and stability under varying environmental conditions, but also to support the production of fruits with desirable nutritional composition and bioactive compound profiles.

## 3. Materials and Methods

### 3.1. Plant Material

The field experiment was conducted on open-field red, yellow and black-purple raspberry cultivars without permanent protective structures. White nonwoven fabric was used only temporarily to protect the plants during spring frost events. The plantation was established in eastern Poland within the Lublin Upland macroregion (51°11′ N, 21°49′ E). The Lubelskie Voivodeship, in which the plantation was located, is one of the main raspberry-growing regions in Poland. The experiment was conducted in a three-year-old raspberry plantation established on soil classified as Class IIIa according to the Polish soil valuation system. Plants were arranged in rows oriented along the east–west axis, with an inter-row spacing of 3.0 m and a within-row spacing of 0.40 m, corresponding to a planting density of approximately 8000 plants ha^−1^. The plants were maintained as continuous hedgerows, which is a common training system in commercial raspberry production. Crop management was carried out in accordance with integrated pest management principles applied in Poland. Fertilization followed current recommendations for raspberry production and was adjusted to plant nutritional requirements, with the same fertilization scheme applied to all cultivars. Irrigation was provided using a drip irrigation system.

For each cultivar, 15 plants were divided into three replicate groups of five plants. Fruit at full harvest maturity was collected separately from each group during the 2025 season. Fruits collected from the five plants within each replicate were pooled to form one composite biological sample, resulting in three independent biological replicates per cultivar (n = 3). The three biological samples were kept separate throughout freezing, storage, sample preparation, and laboratory analysis.

Immediately before the assays, a 15 g portion from each biological replicate was homogenized separately and in its entirety, without prior thawing, using an H500 laboratory homogenizer (POL-EKO-APARATURA sp.j., Wodzisław Śląski, Poland). Homogenization was performed in three 60 s cycles. Separate aliquots were promptly taken from each of the three homogenates for the individual assays. Samples intended for vitamin C determination were homogenized with the addition of metaphosphoric acid.

The studied red and yellow raspberry cultivars belong to the species *R. idaeus*. The taxonomic and colour classification of the cultivar Heban is not entirely consistent across available sources. In some studies, Heban is assigned to *R. occidentalis* and described as a black raspberry [64] or black-purple-fruited raspberry [48], whereas other sources classify it as a purple raspberry [71,72], which should indicate that it belongs to the species *Rubus* × *neglectus*. Fruit colour may also be perceived differently depending on the stage of ripeness, lighting conditions, and the terminology adopted by individual people assessing, resulting in descriptions such as purple, dark purple or black. Therefore, in the present study, Heban was classified as a cultivar with black-purple fruits belonging to *Rubus* × *neglectus*, while acknowledging the inconsistent taxonomic and colour assignments reported in the literature.

**Figure 2:**
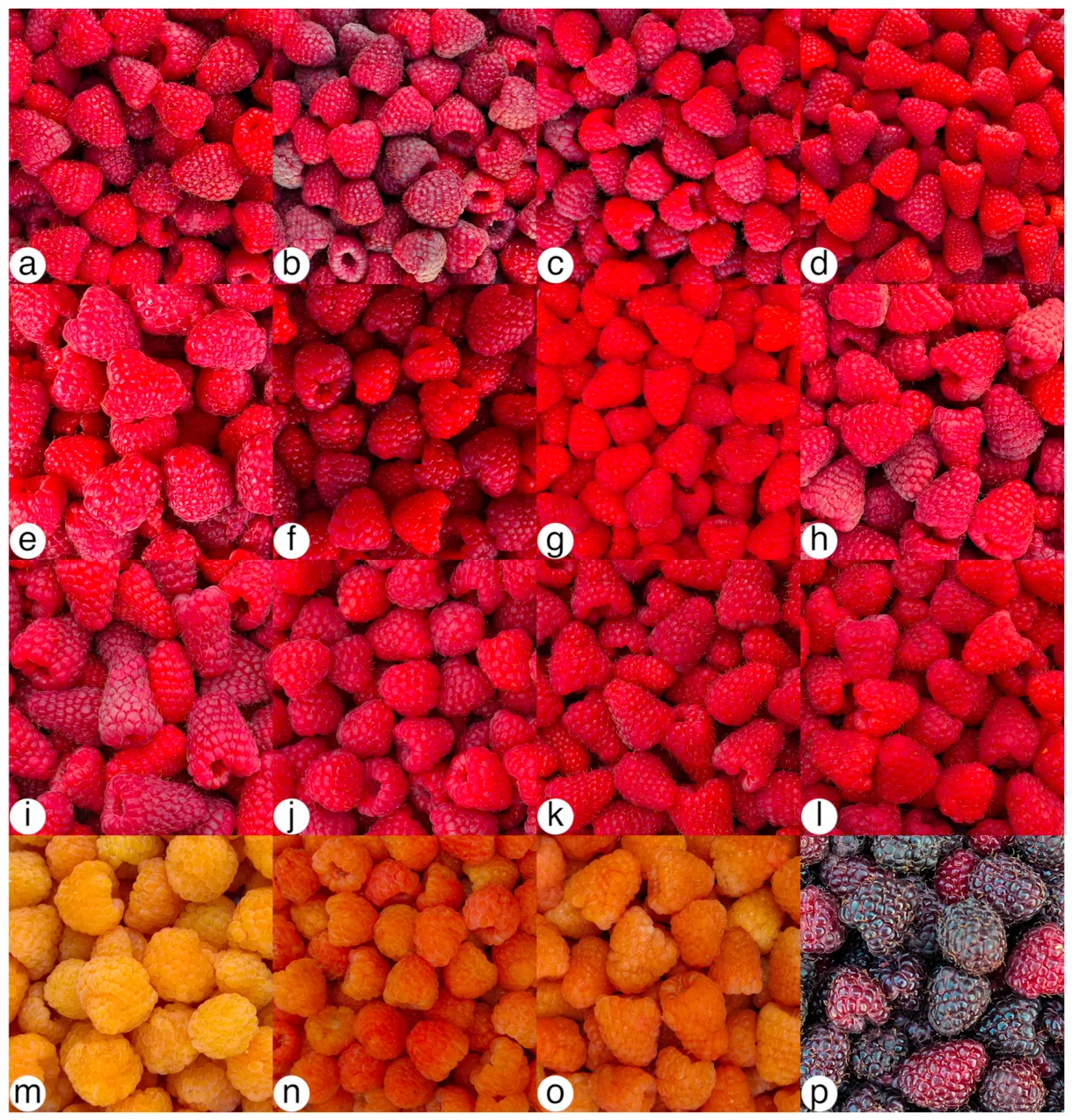
Raspberry fruit: (**a**) Polana; (**b**) Polesie; (**c**) Poemat; (**d**) Polonez; (**e**) Poranek; (**f**) Delniwa; (**g**) Husaria; (**h**) Enrosadira; (**i**) Przehyba; (**j**) Glen Ample; (**k**) Glen Carron; (**l**) Glen Mor; (**m**) Poranna Rosa; (**n**) Jantar; (**o**) Promyk; (**p**) Heban.

Four floricane and twelve primocane raspberry cultivars were included in the study (Table 6). The selected cultivars were chosen based on their importance in contemporary raspberry production in Poland, as well as their suitability for both fresh consumption and processing. Additionally, the research included yellow and black-purple raspberry varieties, which may gain significant popularity in the future. Floricane cultivars were grown with four canes left per shrub, and primocane cultivars with seven. Figure 2 shows the raspberry fruits included in the study.

### 3.2. Analyses

#### 3.2.1. Chemicals

Buffer solutions Hamilton DURACAL (pH 4.01, pH 7.00, pH 9.21), metaphosphoric acid, trisodium phosphate dodecahydrate, L-cysteine, ascorbic acid ≥ 99.0%, HPLC-grade caffeic acid, DPPH, Trolox, and analytical standards of 4-hydroxybenzoic acid, caffeic acid, vanillic acid, syringic acid, ferulic acid, and sinapic acid were purchased from Sigma-Aldrich (Poznań, Poland). Orthophosphoric acid was supplied by Chempur (Piekary Śląskie, Poland). Sodium hydroxide (0.1 mol/L solution) was obtained from Alfachem (Poznań, Poland). The phosphomolybdotungstic reagent was purchased from VWR Chemicals (Gdańsk, Poland). Sodium carbonate, leather powder, pyrogallol, Folin–Ciocalteu reagent, methanol, 10% aqueous solution of disodium carbonate, acetone, ethyl acetate, aluminum chloride, methenamine, glacial acetic acid, hydrochloric acid, ethanol, acetonitrile were supplied by POCH (Gliwice, Poland).

#### 3.2.2. Dry Matter

The dry matter (SM) content was determined in accordance with the PN-A-75101/03:1990 method [73]. An open weighing dish containing 3 g of sand and a glass rod was dried in a Memmert analytical oven (Memmert USA, LLC, Eagle, WI, USA) at 105 °C for 1 h. It was then closed, cooled in a desiccator, and weighed using a Radwag analytical balance AS 310 (RADWAG Wagi Elektroniczne, Radom, Poland). Approximately 3 g of thawed raspberry fruits were added to the dish, mixed with the sand using a glass rod, the dish was closed, and then weighed. The open dishes were dried at 105 °C for 3 h. After drying, the dish was closed, cooled in a desiccator, and weighed. The dry matter content SM (%) was calculated according to Equation (1).

(1) $$\mathrm{SM}=\left(\frac{m_{2}-m}{m_{1}-m}\right)\cdot 100\%$$

where

m—mass of the weighing dish with sand and glass rod (g);

m_1_—mass of the weighing dish with sand, glass rod, and sample before drying (g);

m_2_—mass of the weighing dish with sand, glass rod, and sample after drying (g).

#### 3.2.3. Total Acidity

Total acidity (TA) was determined according to the PN–90/A–75101/04 method [74]. In a 250 mL beaker, 25 g of thawed raspberry fruits were weighed on a Radwag AS 310 laboratory balance), approximately 100 mL of distilled water was added, and the mixture was brought to a boil. The solution was cooled and quantitatively transferred to a 250 mL volumetric flask, made up to the mark, and left to stand for 15 min. The contents of the flask were filtered through filter paper into a dry flask. An aliquot of 10 to 25 mL of the filtrate was pipetted into a 150 mL beaker and diluted to 50 mL with distilled water. The electrodes of a Mettler-Toledo S20-K SevenEasy laboratory pH meter (Mettler-Toledo (Schweiz) GmbH, Greifensee, Switzerland) were immersed in the tested solution, a magnetic stirrer MS 11 HS (Wigo, Pruszków, Poland) was started, and the solution was titrated with 0.1 mol/L sodium hydroxide under continuous stirring to pH 8.1 ± 0.2. Total acidity, expressed as malic acid (g/100 g FW), was calculated according to Equation (2).

(2) $$\mathrm{TA}=\frac{V\cdot N\cdot K\cdot V_{0}\cdot 100}{V_{1}\cdot m}$$

where

V—volume of sodium hydroxide solution used for titration (mL);

N—molarity of the sodium hydroxide solution;

V_0_—volume to which the sample was diluted (mL);

V_1_—volume of the filtrate taken for titration (mL);

M—mass of the sample (g);

K = 0.067—coefficient for converting the result to malic acid.

#### 3.2.4. Total Soluble Solids

Total soluble solids (TSS) were determined in juice obtained by gently pressing the thawed raspberry fruits using a digital handheld refractometer ATAGO PAL-1 (ATAGO CO., Ltd., Tokyo, Japan) [75]. The sample was applied directly to the refractometer prism, and the results were expressed as °Brix.

#### 3.2.5. TSS/TA Ratio

The total soluble solids to titratable acidity ratio (TSS/TA ratio) was calculated as TSS by TA (Equation (3)).

(3) $$\mathrm{TSS}/\mathrm{TA ratio}=\frac{\mathrm{TSS}}{\mathrm{TA}}$$

#### 3.2.6. Vitamin C

Vitamin C (VC) was determined using the HPLC method according to EN 14130:2003 [76]. The liquid chromatograph (Shimadzu, Kyoto, Japan) was equipped with a DGU-20A3 mobile phase degasser, two LC-20AD pumps, an SIL-20ACHT autosampler, a CTO-20AC column oven, an SPD-M20A photodiode array detector, a CBM-20A communication module, and LC Solution software version 1.25 SP5. 15 g of thawed raspberries were weighed using a Radwag AS 310 laboratory balance and were extracted with metaphosphoric acid at a concentration of 20 g/L. After extraction, the sample was reduced. To 20 mL of extract, 10 mL of cysteine solution (40 g/L) was added, followed by trisodium phosphate solution (200 g/L) to adjust the pH to 7.0–7.2. The mixture was stirred for 5 min, and then the pH was lowered to 2.5–2.8 using a concentrated metaphosphoric acid solution (200 g/L). The volume was made up to 50 mL. After mixing, the solution was filtered through a fluted filter paper and then through a SimplePure PTFE syringe filter (Membrane Solutions, Auburn, WA, USA) with a pore size of 0.45 µm. Ascorbic acid was determined using an isocratic method on a Gemini C18 chromatographic column (Phenomenex, Torrance, CA, USA) (150 mm × 4.4 mm, particle size 5 µm). The mobile phase was orthophosphoric acid solution at pH 2.8, with a flow rate of 0.7 mL/min. The column temperature was 20 °C. The injection volume was 20 µL, and the analysis time was 20 min. Identification and quantification of ascorbic acid were performed using a photodiode array detector at 245 nm. Quantification was carried out using the external standard method. A standard solution at a concentration of 0.1 mg/mL was prepared by dissolving ascorbic acid in orthophosphoric acid at pH 2.8. Working standard solutions were prepared by diluting the stock solution with orthophosphoric acid at pH 2.8. The vitamin C content (mg/100 g FW) was calculated using Equation (4).

(4) $$\mathrm{VC}=\frac{Q\cdot F\cdot V\cdot 100}{m\cdot 1000}$$

where

Q—vitamin C concentration read from the calibration curve (µg/mL);

V—total volume of the sample solution after extraction (50 mL);

F—dilution factor at the reduction stage (2.5);

M—sample mass (g);

1000—conversion factor from micrograms to milligrams;

100—factor to express content per 100 g of sample.

#### 3.2.7. Tannins

Tannins (TAN) were determined according to the method described in the Polish Pharmacopoeia (2002) [77]. Into a 250 mL round-bottom flask, about 1 g of raspberries was weighed, 150 mL of water was added, and the mixture was extracted in a boiling water bath MLL 547 (AJL Electronics, Kraków, Poland) for 30 min. The mixture was then cooled under running water, quantitatively transferred to a 250 mL volumetric flask, and made up to volume with water. After complete sedimentation of the solid phase, the clear supernatant was filtered, discarding the first 50 mL of eluate, to obtain the stock solution.

Five milliliters of the stock solution were diluted with water to 25 mL in a volumetric flask. Then, 2 mL of this solution were transferred to a 25 mL volumetric flask, and 1 mL of phosphomolybdotungstic reagent and 10 mL of water were added. The mixture was made up to volume with sodium carbonate solution (290 g/L). It was incubated for 30 min, and the absorbance (A1) was measured using a UV-VIS 1800 spectrophotometer (Shimadzu, Kyoto, Japan) at a wavelength of 760 nm against distilled water.

To 10 mL of the stock solution, 0.10 g of standardized leather powder was added. The mixture was shaken for 1 h, after which the solid phase was removed by filtration. Five milliliters of the filtrate were diluted with water to 25 mL. To 2 mL of this solution, 1 mL of phosphomolybdotungstic reagent and 10 mL of water were added, and the mixture was made up to 25 mL with sodium carbonate solution (290 g/L). After 30 min of incubation, the absorbance (A2) was measured.

Immediately before measurement, a pyrogallol standard solution was prepared by dissolving 50 mg of pyrogallol in distilled water and diluting to 100 mL. Then, 5 mL of this solution was further diluted with distilled water to 100 mL. Two milliliters of the standard solution were taken, 1 mL of phosphomolybdotungstic reagent and 10 mL of distilled water were added, and the mixture was made up to 25 mL with sodium carbonate solution (290 g/L). After 30 min, the absorbance (A3) was measured. The tannin content (%) expressed as pyrogallol was calculated using Equation (5).

(5) $$\mathrm{TAN}=\frac{62.5\cdot (A_{1}-A_{2})\cdot m_{2}}{A_{3}\cdot m_{1}}$$

where

A1—absorbance of the total polyphenolic fraction in the test solution;

A2—absorbance of the polyphenolic fraction not exhibiting affinity to leather powder;

A3—absorbance of the reference pyrogallol solution;

m1—mass of the sample (g);

m2—mass of pyrogallol (g);

62.5—dilution and concentration conversion factor in the measurement system.

#### 3.2.8. Total Phenolic Compounds

The determination of Total phenolic compounds (TPC) was performed according to the modified method of Singleton and Rossi (1965) [78]. Approximately 1 g of raspberries was weighed into a round-bottom flask using a Radwag AS 310 balance, 40 mL of methanol was added, and extraction was carried out in a water bath MLL 547 (AJL Electronics, Kraków, Poland) for 30 min. The sample was filtered into a 50 mL volumetric flask and made up to volume with methanol.

Into a 25 mL volumetric flask, the following were added: 1 mL of extract, 2 mL of methanol, 10 mL of distilled water, and 2 mL of Folin–Ciocalteu reagent. After 3 min, 1 mL of a 10% disodium carbonate solution was added, mixed, and the samples were left to stand for 30 min. After this time, the volume was adjusted to the mark with deionized water, and the absorbance was measured using a UV–VIS spectrophotometer (Shimadzu, Kyoto, Japan) at a wavelength of λ = 750 nm against a blank.

A blank sample was prepared. Into a 25 mL volumetric flask, the following were added: 2 mL of methanol, 10 mL of distilled water, and 2 mL of Folin–Ciocalteu reagent. After 3 min, 1 mL of a 10% disodium carbonate solution was added, mixed, and the samples were left to stand for 30 min. After this time, the volume was adjusted to the mark with deionized water and measured.

A stock solution of caffeic acid at a concentration of 1 mg/mL was prepared by dissolving 50 mg of caffeic acid in 50 mL of methanol. Into five 25 mL volumetric flasks, the following volumes of the stock solution were pipetted: 0.01, 0.03, 0.05, 0.07, and 0.1 mL. Then, 2 mL of methanol, 10 mL of water, and 2 mL of Folin–Ciocalteu reagent were added. After 3 min, 1 mL of a 10% disodium carbonate solution was added and mixed. The samples were left to stand for 30 min, then filled to the mark with deionized water and measured. The obtained solutions corresponded to the following caffeic acid concentrations: 0.0004, 0.0012, 0.0020, 0.0028, and 0.0040 mg/mL. The concentration of phenolic compounds (mg CAE/100 g FW), expressed as caffeic acid equivalents (CAE), was calculated using Equation (6).

(6) $$\mathrm{TPC}=\frac{c\cdot 125000}{V\cdot m}$$

where

c—concentration of caffeic acid equivalents in the final 25 mL reaction mixture, determined from the calibration curve (mg/mL);

V—volume of the fruit extract aliquot used for the assay (mL);

m—mass of fresh fruit used for extraction (g).

The factor 125,000 accounts for the 25 mL final reaction volume, the 50 mL total extract volume, and conversion to 100 g of fresh fruit.

#### 3.2.9. Flavonoids

Flavonoids (TFC) were determined according to the method described in the Polish Pharmacopoeia (2002) [79]. A 1.200 g sample of fresh raspberries was weighed using a Radwag AS 310 balance and transferred to a round-bottom flask. Then, 20 mL of acetone, 2 mL of hydrochloric acid (c = 281 g/L), and 1 mL of methenamine solution (c = 5 g/L) were added, and the mixture was maintained in a boiling water bath MLL 547 (AJL Electronics, Kraków, Poland) for 30 min. The liquid phase was filtered through a cellulose wool filter into a 100 mL volumetric flask.

The solid residue along with the filter was quantitatively transferred back into the round-bottom flask, 20 mL of acetone was added, and the extraction was repeated under reflux for 10 min. The extraction procedure was repeated once more in the same manner. The combined acetone extracts were filtered into the same volumetric flask and diluted to 100 mL with solvent.

A 20 mL portion of the obtained acetone extract was transferred to a separatory funnel, diluted with 20 mL of distilled water, and subjected to liquid–liquid extraction with ethyl acetate in portions: 1 × 15 mL and 3 × 10 mL. The separated organic phases were combined, washed twice with distilled water (2 × 40 mL), filtered through anhydrous sodium sulfate into a 50 mL volumetric flask, and diluted to volume with ethyl acetate (stock solution).

Into a 25 mL volumetric flask, 10 mL of the stock solution was introduced, 2 mL of aluminum chloride solution (c = 20 g/L) was added, and the mixture was diluted to the final volume with a mixture of glacial acetic acid (d = 1.02 kg/L) and methanol in a 1:19 volume ratio. A blank sample was prepared. Into a 25 mL volumetric flask, 10 mL of the stock solution was introduced and diluted to volume with a mixture of acetic acid and methanol (1:19), omitting the complexing agent, aluminum chloride. Both solutions were incubated in the dark for 45 min. The absorbance (A) of the test solution was measured using a UV-VIS 1800 spectrophotometer (Shimadzu, Kyoto, Japan) at a wavelength of λ = 425 nm against the blank. The total flavonoid content (mg RE/100 g FW), expressed as rutin (RE), was calculated from Equation (7).

(7) $$\mathrm{TFC}=\frac{A\cdot k}{m\cdot 1000}$$

where

A—absorbance of the tested solution at wavelength λ = 425 nm;

m—mass of the sample (g);

k = 2.06—conversion factor for rutin.

#### 3.2.10. Total Anthocyanin Content

Total anthocyanin content (TAC) was determined according to the method of the Australian Wine Research Institute (2021) with modifications [80]. Approximately 1.0 g ± 0.1 of raspberries was weighed into a 10 mL centrifuge tube using a Radwag analytical balance AS 310. To the weighed sample, 10 mL of acidified 50% *v*/*v* ethanol solution was added, after which the tube was tightly sealed. Extraction was carried out for 1 h on a rotary shaker, protecting the samples from direct light exposure.

After extraction, the samples were centrifuged at approximately 3500 rpm for 5 min using a Centrifuge 5415R (Eppendorf AG, Hamburg, Germany). The supernatant was carefully decanted into a graduated cylinder, and the final extract volume was measured with an accuracy of 0.2 mL. The obtained extract volume was used in the calculation of the final anthocyanin content.

A 1 mL aliquot of the extract was transferred to a test tube, and 10 mL of 1 M HCl was added. The total volume after dilution was 11 mL; therefore, the dilution factor was 11. The prepared solution was incubated in the dark at room temperature for at least 1 h, but not longer than 4 h. The UV-VIS 1800 spectrophotometer (Shimadzu, Kyoto, Japan) was set to a wavelength of 520 nm. The instrument was zeroed against 1 M HCl using a quartz cuvette with a 10 mm optical path length. The absorbance of the diluted extract was then measured at 520 nm. Total anthocyanin content (mg/100 g FW) was calculated according to Equation (8):

(8) $$\mathrm{TAC}=\frac{A_{520}\cdot \mathrm{DF}\cdot V\cdot 1000\cdot 100}{500\cdot 100\cdot m}$$

where

A_520_—absorbance of the diluted extract measured at 520 nm;

DF—dilution factor = 11;

V—final volume of the extract (mL);

1000—conversion factor from g to mg;

100—conversion factor from g to 100 g;

500—specific absorption coefficient used in the method;

100—factor resulting from conversion of percentage concentration;

m—mass of the sample used for extraction (g).

#### 3.2.11. Antioxidant Activity by the FRAP Method

The antioxidant activity was measured using the FRAP method with the Merck Ferric Reducing Antioxidant Power (FRAP) Assay Kit (MAK509-1KT) according to the manufacturer’s instructions [81] without modification of the assay procedure. A calibration curve was prepared using the Fe^2+^ standard supplied with the kit. Results obtained for the extracts were adjusted for dilution and extraction volume and expressed as µmol Fe^2+^ equivalents per gram of fresh fruit (µmol Fe^2+^ eq./g FW).

#### 3.2.12. Antioxidant Activity by the DPPH Method

The antioxidant activity was determined using the DPPH method according to a modified procedure described by Brand-Williams et al. (1995) [82]. The DPPH radical solution was prepared by dissolving 2.4 mg of DPPH in 100 mL of methanol. The prepared solution showed an absorbance of 0.6 relative to methanol, measured with a spectrophotometer at a wavelength of 515 nm at 22 °C. The dark purple solution was stored at a temperature below 8 °C.

The 1 g sample of raspberries was weighed, 20 mL of methanol was added, and the mixture was thoroughly mixed. After 2 h, the obtained extract was decanted from the sample and used for analysis. For the assay, 0.195 mL of the DPPH radical solution was transferred into a well of a microplate reader TECAN Infinite 200 Pro (Tecan Austria GmbH, Grödig, Austria), followed by the addition of 0.005 mL of the appropriately diluted sample solution. The mixture was shaken for 10 s. The absorbance of the radical solution was measured before adding the sample, and then the decrease in absorbance at 515 nm was measured after 15 min against pure methanol at 22 °C. Antioxidant activity was expressed as Trolox equivalent antioxidant capacity (TEAC) and reported in µmol TE/g FW. To prepare the calibration curve, a methanolic Trolox solution with a concentration of 0.156 mg/mL was prepared. This solution was diluted 1-, 4-, 6-, 8-, and 10-fold to obtain working solutions, which were used to construct the calibration curve. Measurements for the calibration curve were performed by placing 0.195 mL of DPPH solution and 0.005 mL of the appropriately diluted Trolox solution into a microplate well. The mixture was shaken for 10 s. Absorbance was measured at 515 nm after 15 min against pure methanol at 22 °C.

#### 3.2.13. Phenolic Acids

Selected phenolic acids were analyzed using the HPLC method [83]. The liquid chromatograph (Shimadzu, Kyoto, Japan) was equipped with a mobile phase degasser DGU-20A3, pump A—LC-20AD, pump B—LC-20AD, autosampler SIL-20ACHT, column oven CTO-20AC, UV-VIS DAD detector SPD-M20A, communication module CBM20A, and LC Solution software.

In total, 1 g of raspberries was placed into an Eppendorf centrifuge tube, 25 mL of methanol was added, and the mixture was extracted for 30 min using a GFL laboratory shaker model 3017 (GFL Gesellschaft für Labortechnik mbH, Burgwedel, Germany). Then, 1 mL of the extract was transferred to a centrifuge tube, 100 µL each of Carrez I and Carrez II solutions were added, and the mixture was shaken. Subsequently, the sample was centrifuged for 10 min using a Centrifuge 5415R Eppendorf (Warsaw, Poland). The upper layer of the solution was collected and filtered through a syringe filter with a PTFE membrane (0.22 µm pore size). The injection volume was 20 µL.

Chromatographic separation was performed in a reversed-phase system on a Hypersil Gold C18 column (Thermo Fisher Scientific, Waltham, MA, USA;, 250 mm × 4.6 mm, particle size 5 µm). The mobile phase consisted of HPLC-grade water with 0.1% glacial acetic acid (phase A) and 50% acetonitrile (phase B). Separation was carried out using a gradient system of two eluents: 1% (*v*/*v*) acetic acid and 50% (*v*/*v*) acetonitrile: 0.01–11 min, 92% A and 8% B; 11–41 min, 70% A and 30% B; 41–56 min, 60% A and 40% B; 56–71 min, 92% A and 8% B, at a flow rate of 0.8 mL/min. Eluent solutions were prepared in deionized water and then degassed in an ultrasonic bath with Sonic-5 POLSONIC Palczyński Sp. J., Warsaw, Poland).

The wavelength was selected based on the UV absorption maxima of individual phenolic compounds—mainly 280 nm. Qualitative analysis was performed based on determined retention times and recorded UV-VIS absorption spectra of standards. The content of phenolic acids was expressed in µg/g FW. As part of the validation of the method for determining selected phenolic compounds in raspberries, the following validation parameters were established: limit of detection (LOD), limit of quantification (LOQ) (Table 7), repeatability, and recovery (Table 8). Representative HPLC-PDA chromatograms obtained during the analysis of selected phenolic acids in raspberry fruit samples are provided in the Supplementary Materials (Figures S1–S6).

### 3.3. Statistical Analysis

Statistical analyses were conducted using RStudio (Posit PBC, Boston, MA, USA) version 2025.9.2.418, using the R programming language (version 4.4.0). Prior to analysis, the assumptions of the model were verified. Normality of residuals was assessed using the Shapiro–Wilk test, whereas homogeneity of variances was evaluated with Levene’s test. No significant violations of these assumptions were detected. One-way analysis of variance (ANOVA) was applied to determine differences among treatments. When significant effects were observed, mean separation was performed using Tukey’s honestly significant difference (HSD) test. Each biological replicate was analyzed separately, and the resulting three values per cultivar were used as independent observations in the statistical analyses. The results are presented as homogeneous groups indicated by lowercase letters; different lowercase letters within the same column indicate statistically significant differences, whereas identical letters indicate no statistically significant differences (*p* ≤ 0.05). For each dataset, *p*-values obtained from the F-test are reported, and statistical significance was accepted at *p* ≤ 0.05.

Relationships among parameters were evaluated using Pearson correlation analysis across all cultivars. The analysis included three replicates for each of the sixteen cultivars, resulting in forty-eight observations (n = 48) for each pairwise correlation. The resulting correlation matrix was presented in the form of a heatmap.

## 4. Conclusions

The present study demonstrates substantial cultivar-dependent variation in raspberry fruit quality, bioactive compound composition, and in vitro antioxidant capacity. The observed variability in physicochemical traits and phenolic composition, together with the relationships identified with antioxidant capacity, suggests that antioxidant properties are associated with multiple bioactive compound groups rather than a single dominant component. These findings highlight the value of integrating physicochemical and compositional analyses when evaluating raspberry fruit quality and indicate the potential usefulness of cultivar selection for obtaining distinct fruit quality and bioactive compound profiles. Overall, the results contribute to a broader understanding of cultivar-dependent variation in raspberry fruit composition; however, multi-year and multi-location studies are required to determine the stability of the observed differences and to support broader recommendations for breeding, processing, or other applied uses.

## Figures and Tables

**Table 1:** Fruit quality parameters.

Fruit Color	Cultivar	Dry Matter (%)	Total Soluble Solids (°Brix)	Titratable Acidity (g/100 g FW)	TSS/TA Ratio
Red	Polana	16.48 ± 0.25 b	8.97 ± 0.25 def	2.64 ± 0.07 a	3.40 ± 0.01 i
	Polesie	14.63 ± 0.19 d	9.63 ± 0.21 cd	1.90 ± 0.06 defg	5.07 ± 0.22 fgh
	Poemat	13.57 ± 0.17 e	10.00 ± 0.36 c	2.17 ± 0.07 bc	4.62 ± 0.31 h
	Polonez	16.26 ± 0.22 b	11.37 ± 0.31 b	1.52 ± 0.05 i	7.49 ± 0.43 b
	Poranek	15.53 ± 0.20 c	9.33 ± 0.21 cde	2.74 ± 0.09 a	3.41 ± 0.18 i
	Delniwa	17.29 ± 0.18 a	12.70 ± 0.30 a	1.96 ± 0.07 def	6.49 ± 0.37 c
	Husaria	13.04 ± 0.17 ef	12.57 ± 0.25 a	1.72 ± 0.06 gh	7.31 ± 0.24 b
	Enrosadira	12.63 ± 0.17 f	9.83 ± 0.25 c	1.53 ± 0.05 i	6.43 ± 0.13 cd
	Przehyba	15.20 ± 0.20 c	9.93 ± 0.06 c	1.79 ± 0.05 efg	5.55 ± 0.18 ef
	Glen Ample	11.97 ± 0.18 g	9.37 ± 0.15 cde	1.97 ± 0.06 de	4.76 ± 0.19 gh
	Glen Carron	13.32 ± 0.17 e	8.50 ± 0.27 f	1.78 ± 0.06 fg	4.78 ± 0.05 gh
	Glen Mor	15.39 ± 0.21 c	8.63 ± 0.15 ef	2.26 ± 0.07 b	3.82 ± 0.19 i
Yellow	Poranna rosa	8.36 ± 0.14 i	11.70 ± 0.10 b	2.03 ± 0.06 cd	5.77 ± 0.18 de
	Jantar	14.53 ± 0.17 d	12.57 ± 0.21 a	2.34 ± 0.07 b	5.37 ± 0.16 efg
	Promyk	14.29 ± 0.19 d	13.30 ± 0.44 a	1.59 ± 0.05 hi	8.37 ± 0.12 a
Black-purple	Heban	11.15 ± 0.17 h	9.83 ± 0.32 c	1.41 ± 0.05 i	6.98 ± 0.09 bc
	*p*-value	<0.001	<0.001	<0.001	<0.001

**Table 2:** Bioactive compounds.

Fruit Color	Cultivar	Vitamin C (mg/100 g FW)	Tannins (% FW)	Total Phenolic Compounds (mg CAE/100 g FW)	Flavonoids (mg RE/100 g FW)	Anthocyanins (mg/100 g FW)
Red	Polana	19.53 ± 0.72 d	0.039 ± 0.001 de	84.58 ± 2.11 de	3.15 ± 0.03 de	26.14 ± 0.70 f
	Polesie	18.29 ± 0.62 cd	0.034 ± 0.001 fg	109.93 ± 3.94 b	1.32 ± 0.05 i	16.99 ± 0.47 i
	Poemat	14.11 ± 0.65 h	0.027 ± 0.001 hi	80.93 ± 2.27 d	1.43 ± 0.03 hi	52.32 ± 1.07 b
	Polonez	17.35 ± 0.64 ef	0.024 ± 0.001 ij	59.80 ± 1.97 g	3.93 ± 0.15 b	14.49 ± 0.42 j
	Poranek	20.12 ± 0.78 bd	0.035 ± 0.001 efg	91.94 ± 2.57 c	2.60 ± 0.10 f	23.93 ± 0.72 g
	Delniwa	22.27 ± 0.77 c	0.035 ± 0.001 efg	55.31 ± 1.30 g	0.90 ± 0.04 j	31.64 ± 0.65 d
	Husaria	18.58 ± 0.71 cd	0.040 ± 0.001 d	46.18 ± 1.61 h	3.18 ± 0.11 d	8.19 ± 0.28 l
	Enrosadira	18.70 ± 0.72 cd	0.017 ± 0.001 k	61.11 ± 1.99 fg	2.91 ± 0.09 e	26.77 ± 0.69 f
	Przehyba	32.01 ± 1.16 a	0.072 ± 0.002 b	61.20 ± 1.97 fg	1.49 ± 0.06 hi	29.28 ± 0.69 e
	Glen Ample	24.91 ± 0.89 b	0.037 ± 0.001 def	105.20 ± 3.54 b	0.66 ± 0.03 k	39.35 ± 0.83 c
	Glen Carron	15.21 ± 0.64 gh	0.091 ± 0.003 a	86.79 ± 2.42 de	0.92 ± 0.02 j	12.18 ± 0.34 k
	Glen Mor	21.84 ± 0.83 bc	0.074 ± 0.002 b	87.35 ± 2.37 de	2.19 ± 0.07 g	20.42 ± 0.57 h
Yellow	Poranna rosa	11.54 ± 0.53 i	0.047 ± 0.001 c	68.04 ± 2.10 ef	3.52 ± 0.13 c	2.51 ± 0.11 m
	Jantar	22.70 ± 0.72 c	0.037 ± 0.001 def	54.63 ± 1.61 g	2.89 ± 0.06 e	2.63 ± 0.11 m
	Promyk	14.22 ± 0.53 h	0.031 ± 0.002 gh	72.22 ± 2.15 e	1.61 ± 0.06 h	1.27 ± 0.08 m
Black-purple	Heban	6.53 ± 0.33 j	0.020 ± 0.001 jk	128.10 ± 3.77 a	8.89 ± 0.14 a	71.22 ± 1.40 a
	*p*-value	<0.001	<0.001	<0.001	<0.001	<0.001

**Table 3:** Antioxidant activity.

Fruit Color	Cultivar	FRAP (µmol Fe^2+^ eq./g FW)	DPPH (µmol TE/g FW)
Red	Polana	9.59 ± 0.18 ij	3.69 ± 0.08 k
	Polesie	13.60 ± 0.27 defg	8.08 ± 0.17 i
	Poemat	11.24 ± 0.16 h	4.72 ± 0.12 j
	Polonez	13.71 ± 0.21 def	14.47 ± 0.30 f
	Poranek	13.81 ± 0.28 cd	11.93 ± 0.25 g
	Delniwa	9.42 ± 0.25 j	3.13 ± 0.08 k
	Husaria	13.00 ± 0.16 efg	30.04 ± 0.50 a
	Enrosadira	13.12 ± 0.15 defg	22.68 ± 0.38 c
	Przehyba	13.52 ± 0.31 defg	14.19 ± 0.27 f
	Glen Ample	14.10 ± 0.33 c	15.83 ± 0.29 e
	Glen Carron	12.58 ± 0.24 g	11.02 ± 0.21 h
	Glen Mor	12.94 ± 0.25 fg	12.61 ± 0.21 g
Yellow	Poranna rosa	15.31 ± 0.24 b	27.26 ± 0.50 b
	Jantar	10.19 ± 0.15 i	18.74 ± 0.32 d
	Promyk	9.87 ± 0.24 ij	8.31 ± 0.17 i
Black-purple	Heban	30.58 ± 0.43 a	18.20 ± 0.30 d
	*p*-value	<0.001	<0.001

**Table 4:** Comparison of fruit quality and bioactive properties of red-fruited floricane and primocane cultivars.

Parameter	Floricane	Primocane	*p*-Value
Dry matter (%)	13.97 ± 1.45 b	14.93 ± 1.66 a	0.038
Total soluble solids (°Brix)	9.11 ± 0.61 b	10.60 ± 1.42 a	<0.001
Titratable acidity (g/100 g FW)	1.95 ± 0.21 a	2.02 ± 0.45 a	0.475
TSS/TA ratio	4.73 ± 0.64 b	5.53 ± 1.58 a	0.027
Vitamin C (mg/100 g FW)	23.49 ± 6.22 a	18.62 ± 2.32 b	0.001
Tannins (% FW)	0.068 ± 0.020 a	0.031 ± 0.008 b	<0.001
Total phenolic compounds (mg CAE/100 g FW)	85.14 ± 16.17 a	73.72 ± 20.72 b	0.039
Flavonoids (mg RE/100 g FW)	1.31 ± 0.60 b	2.43 ± 1.03 a	<0.001
Anthocyanins (mg/100 g FW)	25.31 ± 10.35 a	25.06 ± 12.80 a	0.942
FRAP (µmol Fe^2+^ eq./g FW)	13.29 ± 0.64 a	12.19 ± 1.77 b	0.006
DPPH (µmol TE/g FW)	13.41 ± 1.84 a	12.34 ± 9.29 a	0.583

**Table 5:** Occurrence of phenolic acids.

	Cultivar	4-Hydroxybenzoic Acid (µg/g FW)	Caffeic Acid (µg/g FW)	Vanillic Acid (µg/g FW)	Syringic Acid (µg/g FW)	Ferulic Acid (µg/g FW)	Sinapic Acid (µg/g FW)
Red	Polana	ND	<LOQ = 0.48	ND	ND	<LOQ = 0.44	0.50
	Polesie	ND	ND	<LOQ = 0.47	ND	<LOQ = 0.44	<LOQ = 0.45
	Poemat	ND	<LOQ = 0.48	<LOQ = 0.47	ND	ND	<LOQ = 0.45
	Polonez	<LOQ = 0.50	ND	<LOQ = 0.47	ND	<LOQ = 0.44	<LOQ = 0.45
	Poranek	<LOQ = 0.50	ND	ND	ND	<LOQ = 0.44	<LOQ = 0.45
	Delniwa	ND	ND	ND	ND	<LOQ = 0.44	<LOQ = 0.45
	Husaria	ND	ND	ND	ND	<LOQ = 0.44	<LOQ = 0.45
	Enrosadira	ND	ND	ND	ND	0.70	<LOQ = 0.45
	Przehyba	ND	ND	ND	ND	<LOQ = 0.44	<LOQ = 0.45
	Glen Ample	ND	ND	ND	ND	<LOQ = 0.44	<LOQ = 0.45
	Glen Carron	ND	ND	ND	ND	<LOQ = 0.44	<LOQ = 0.45
	Glen Mor	ND	ND	ND	ND	<LOQ = 0.44	<LOQ = 0.45
Yellow	Poranna rosa	ND	<LOQ = 0.48	ND	ND	<LOQ = 0.44	0.5
	Jantar	ND	ND	ND	0.50	<LOQ = 0.44	<LOQ = 0.45
	Promyk	ND	ND	ND	ND	<LOQ = 0.44	<LOQ = 0.45
Black-purple	Heban	<LOQ = 0.50	ND	<LOQ = 0.47	ND	<LOQ = 0.44	0.50

**Table 6:** Cultivars.

Species	Fruit Color	Fruiting Type	Cultivar	Country of Origin	Breeding Program
*R. idaeus*	Red	Primocane	Polana	Poland	Sadowniczy Zakład Doświadczalny Brzezna
		Primocane	Polesie	Poland	Sadowniczy Zakład Doświadczalny Brzezna
		Primocane	Poemat	Poland	Sadowniczy Zakład Doświadczalny Brzezna
		Primocane	Polonez	Poland	Sadowniczy Zakład Doświadczalny Brzezna
		Primocane	Poranek	Poland	Sadowniczy Zakład Doświadczalny Brzezna
		Primocane	Delniwa	Poland	Niwa Hodowla Roślin Jagodowych
		Primocane	Husaria	Poland	Niwa Hodowla Roślin Jagodowych
		Primocane	Enrosadira	Italy	Molari Berries & Breeding
		Floricane	Przehyba	Poland	Sadowniczy Zakład Doświadczalny Brzezna
		Floricane	Glen Ample	Scotland	James Hutton Institute
		Floricane	Glen Carron	Scotland	James Hutton Institute
		Floricane	Glen Mor	Scotland	James Hutton Institute
	Yellow	Primocane	Poranna Rosa	Poland	Sadowniczy Zakład Doświadczalny Brzezna
		Primocane	Jantar	Poland	Niwa Hodowla Roślin Jagodowych
		Primocane	Promyk	Poland	Niwa Hodowla Roślin Jagodowych
*R. × neglectus*	Black-purple	Primocane	Heban	Poland	Niwa Hodowla Roślin Jagodowych

**Table 7:** Limit of detection and limit of quantification for the method of determining phenolic compounds in raspberry fruits.

Phenolic Acid	Limit of Detection (LOD) (µg/g)	Limit of Quantification (LOQ) (µg/g)
4-Hydroxybenzoic acid	0.17	0.50
Ferulic acid	0.15	0.44
Caffeic acid	0.16	0.48
Vanillic acid	0.16	0.47
Sinapic acid	0.15	0.45
Syringic acid	0.14	0.42

**Table 8:** Repeatability and recovery for the method of determining phenolic compounds in raspberry fruits.

Phenolic Acid	Standard Deviation (SD)	Recovery (%)	Repeatability (CV)
4-Hydroxybenzoic acid	1.25	92.80	3.57
Ferulic acid	0.98	95.35	2.80
Caffeic acid	1.03	97.40	2.94
Vanillic acid	1.15	94.26	3.29
Sinapic acid	1.02	96.43	2.91
Syringic acid	0.95	94.77	2.71

## Data Availability

The data supporting the findings of this study are included in the article and its Supplementary Materials.

## Supplementary Materials

The following supporting information can be downloaded at https://www.mdpi.com/article/10.3390/molecules31203703/s1. Figure S1: Representative HPLC-PDA chromatogram of selected phenolic acids in fruits of the raspberry cultivar Polana; Figure S2: Representative HPLC-PDA chromatogram of selected phenolic acids in fruits of the raspberry cultivar Poranek; Figure S3: Representative HPLC-PDA chromatogram of selected phenolic acids in fruits of the raspberry cultivar Enrosadira; Figure S4: Representative HPLC-PDA chromatogram of selected phenolic acids in fruits of the raspberry cultivar Poranna rosa; Figure S5: Representative HPLC-PDA chromatogram of selected phenolic acids in fruits of the raspberry cultivar Jantar; Figure S6: Representative HPLC-PDA chromatogram of selected phenolic acids in fruits of the raspberry cultivar Heban.
