# Supplementary material for: Comparative Evaluation of Bioactive Compounds, Antioxidant Capacity, and Quality Traits of Raspberry Cultivars
Source: Molecules. 2026 Oct 15;31(20):3703. doi: 10.3390/molecules31203703 (PMC13707578; doi:10.3390/molecules31203703)
Supplement: Supplementary file 1 [file molecules-31-03703-s001.zip › molecules-4580797-supplementary.pdf]

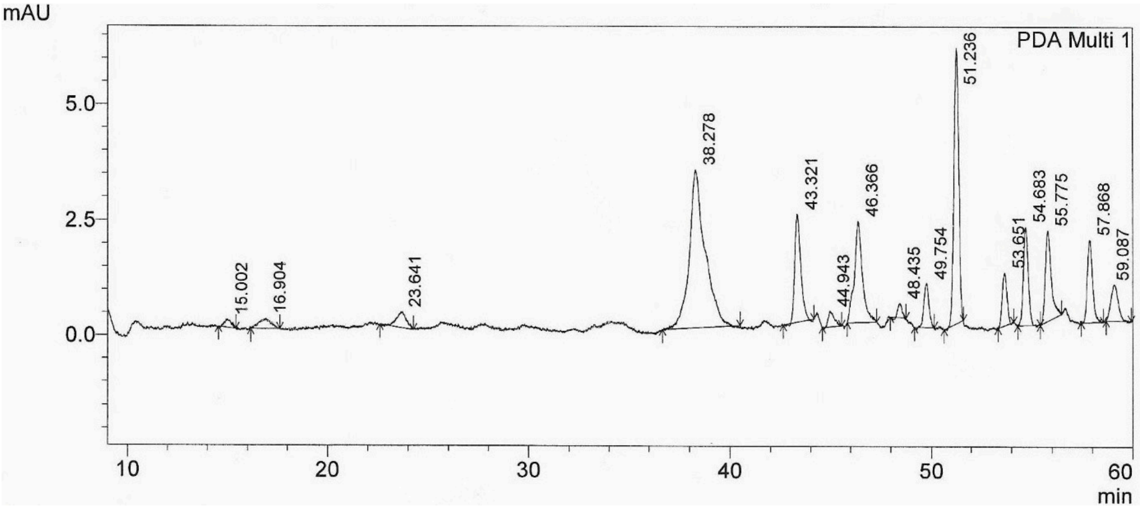

Figure S1. Representative HPLC-PDA chromatogram of selected phenolic acids in fruits of the raspberry cultivar Polana.

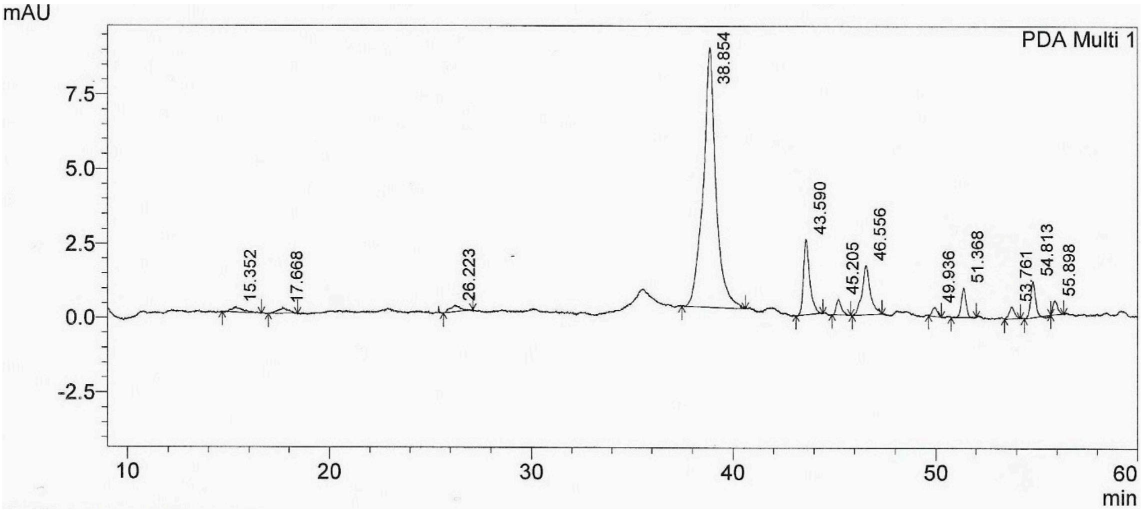

Figure S2. Representative HPLC-PDA chromatogram of selected phenolic acids in fruits of the raspberry cultivar Poranek.

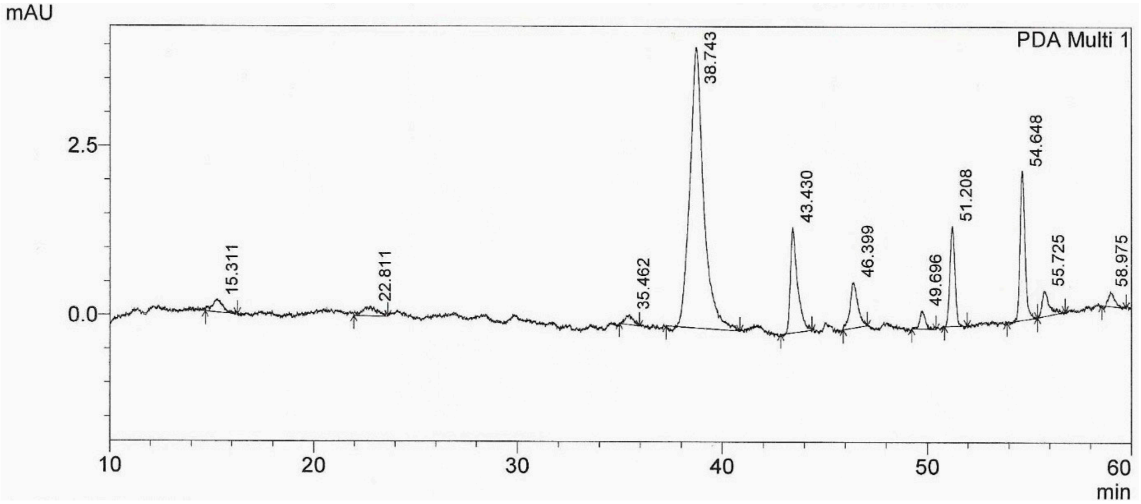

Figure S3. Representative HPLC-PDA chromatogram of selected phenolic acids in fruits of the raspberry cultivar Enrosadira.

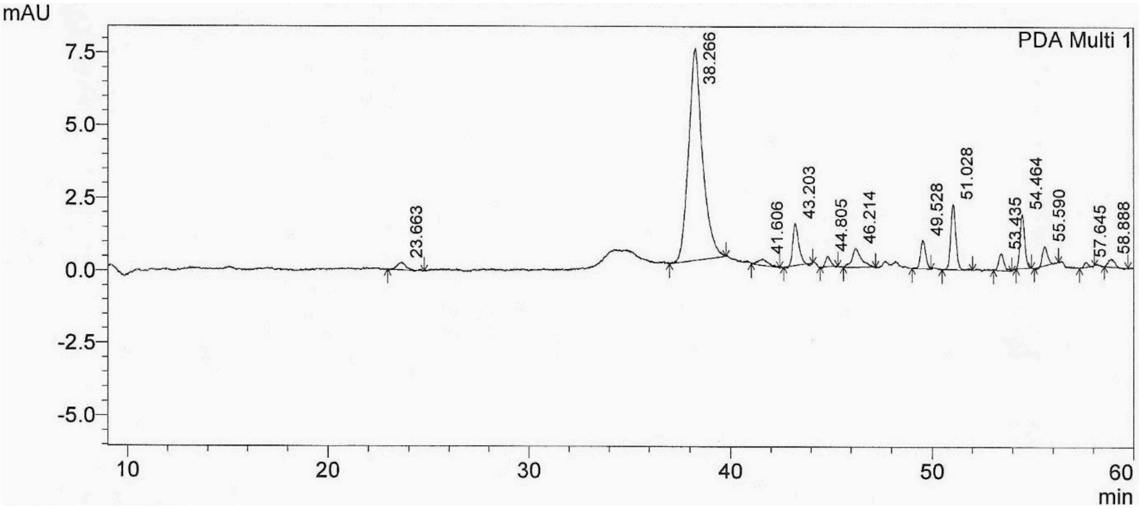

Figure S4. Representative HPLC-PDA chromatogram of selected phenolic acids in fruits of the raspberry cultivar Poranna rosa.

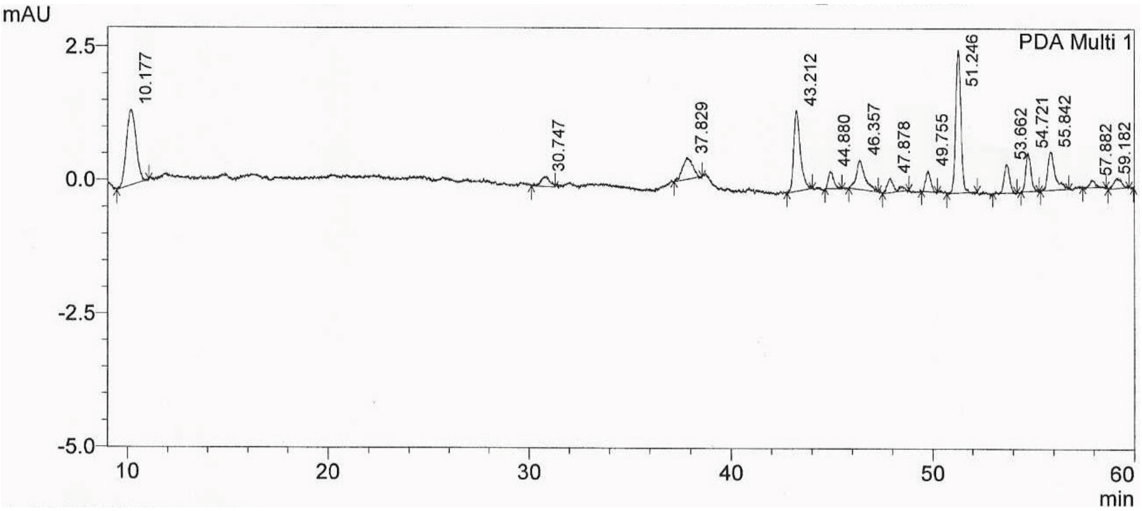

Figure S5. Representative HPLC-PDA chromatogram of selected phenolic acids in fruits of the raspberry cultivar Jantar.

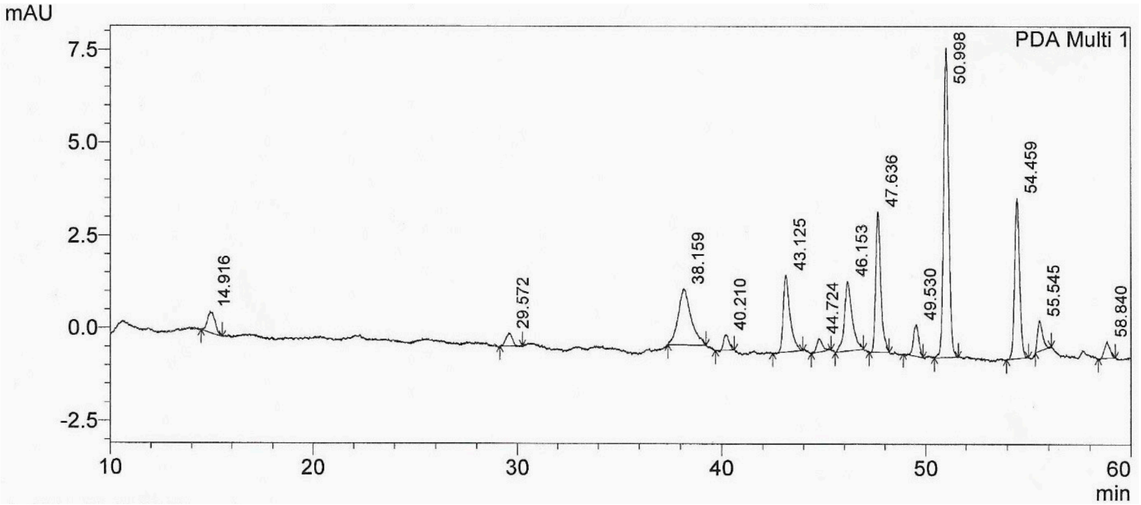

Figure S6. Representative HPLC-PDA chromatogram of selected phenolic acids in fruits of the raspberry cultivar Heban.
